# Experiences of healthcare for people living with multiple sclerosis and their healthcare professionals

**DOI:** 10.1111/hex.13348

**Published:** 2021-09-04

**Authors:** Eluned Price, Robyn Lucas, Jo Lane

**Affiliations:** ^1^ Australian National University Medical School, College of Health and Medicine Australian National University Canberra Australian Capital Territory Australia; ^2^ National Centre for Epidemiology and Population Health, Research School of Population Health, College of Health and Medicine Australian National University Canberra Australian Capital Territory Australia

**Keywords:** healthcare, MS, multiple sclerosis, patient experience, qualitative research, semistructured interviews

## Abstract

**Background:**

Multiple sclerosis (MS) is a chronic inflammatory and neurodegenerative condition of the central nervous system that commonly strikes in young adulthood and has no cure. Many people living with MS (PwMS) will have significant contact with a range of healthcare professionals (HCPs). To achieve optimal health outcomes in MS, it is important to understand factors that contribute to positive or negative healthcare experiences. Previous studies have shown that PwMS want clear communication and in‐depth relationships with their HCPs. However, many studies have lacked qualitative feedback from HCPs.

**Objective:**

This study aimed to investigate healthcare experiences of PwMS and HCPs and identify areas that are working well and areas that could be improved.

**Methods:**

Semistructured interviews with 15 PwMS and 11 HCPs (seven neurologists, four MS nurses) from across Australia were conducted. Interviews were transcribed verbatim and analysed thematically.

**Results:**

Both PwMS and HCPs valued clear communication, recognized uncertainties associated with MS and highlighted the importance of rapport. PwMS focused on decision‐making, understanding roles and expectations, self‐directed management and their needs for support. HCPs discussed issues related to medical management, providing hope and reassurance, barriers to healthcare and multidisciplinary care.

**Conclusion:**

Greater transparency and communication, particularly around the approach to care and the roles played by HCPs, is likely to enhance healthcare experiences and contribute to better health outcomes for PwMS.

**Public Contribution:**

PwMS and HCPs volunteered to be interviewed, and PwMS assisted with the development of interview content and structure.

## 1 INTRODUCTION

Multiple sclerosis (MS) is a chronic neurodegenerative condition involving demyelination in the central nervous system that impacts over 25,000 Australians.^1^, ^2^ The experience of MS is unique to each person living with this condition; however, it typically involves impairment in cognitive, motor and sensory functioning. In Australia, the typical healthcare journey begins with consultation with a primary care physician following the onset of symptoms. For some people who will go on to develop MS, these initial symptoms can be vague, and there will be recurrent visits to the primary care physician before referral for specialist care and investigation. For others, symptom onset will be dramatic, for example loss of vision in one eye, leading to an initial contact with the healthcare system through the emergency department, followed by rapid referral to a specialist neurologist. The latter initiates care, further detailed investigations, for example MRI, and typically maintains the ongoing neurological care of the patient, with variable involvement of the primary care physician. In Australia, subsidized drug treatment can only be provided to people living with MS (PwMS) once the diagnosis of MS is confirmed, typically following a second bout of neurological symptoms. Specialist MS nurses are available in some major cities to provide support to neurologists and PwMS.

There are many factors that contribute to improved outcomes in healthcare including patient education and partnership between a physician and a patient.^3^ As MS is a chronic condition that requires ongoing interaction with the healthcare system, it is particularly important to understand the factors that contribute to optimal health outcomes. For example, recent evidence indicates that, for PwMS, previous healthcare experiences heavily influence future decisions to seek care for MS‐related matters.^4^ Further, although previous studies highlight an overall level of satisfaction with care, they reveal a greater need for informative communication during the early stages of diagnosis and management, as well as greater input from the PwMS in decision‐making.^5^, ^6^

It is not uncommon in MS to have a significant delay between the onset of symptoms and diagnosis of MS, and inadequate provision of support and information during this time is reported.^7^ While treatment and understanding of MS have improved over the past few decades, it is unclear whether this has led to objective improvements in the diagnostic experience for PwMS.^7^, ^8^ A recent meta‐synthesis of qualitative research into the overall experiences of PwMS showed that many individuals described lack of information and personalized advice at the time of diagnosis.^9^ These previous studies have heavily focused on the experiences of PwMS.

There is limited literature outlining the experiences of healthcare professionals (HCPs) in MS healthcare, with these studies highlighting more clinical concerns, such as treatment choices, management of side effects and alignment of care with the personal goals and preferences of PwMS.^10^, ^11^ However, concern has been raised regarding discrepancies between PwMS and HCPs in expectations of care.^12^ A systematic review of studies into HCP–PwMS interactions found that PwMS often felt uninformed following appointments, and felt a lack of depth in the relationship with their HCPs.^13^ However, input from HCPs was underrepresented in this study. It is noteworthy that most previous studies have been quantitative; however, qualitative research may be particularly suited to gaining a deeper understanding of healthcare experiences from different perspectives.

Evaluation of healthcare for PwMS in Australia should be informed by qualitative research that includes the experiences of both PwMS and HCPs, to assess what is currently working well and to identify what needs to be addressed. This qualitative study aims to examine the following research questions:

1. What is the experience of MS healthcare for both PwMS and their HCPs?
2. For PwMS and their HCPs, what works well and are there any areas in healthcare that could be changed?

## 2 METHODS

### 2.1 Study design

This was an explorative qualitative study using purposive sampling to interview 15 PwMS and 11 HCPs specializing in MS.

### 2.2 Interviews

We recruited participants from among those who had been previously involved in research with the research team, using personal invitations and circulation of study flyers. This study was approved by the researchers' university Human Research Ethics Committee, and written consent was obtained from all participants. To be eligible for the study, PwMS had to be over 18 years of age with a diagnosis of MS. HCPs who had extensive experience working with PwMS were invited to participate in the study, including neurologists and MS nurses. It was expected that saturation (i.e., no new insights obtained in the interviews) would be reached within 10–12 interviews if the purposive samples were relatively homogeneous.^14^

Before the interview, PwMS completed a 10‐min online questionnaire using Qualtrics^15^ to collect demographic and clinical characteristics.

The first four interviews were conducted face to face and the remainder by telephone due to local Government regulations during the SARS‐CoV‐2 pandemic. The interview questions were based on a top‐down (theoretical) approach using findings from current research literature and a bottom‐up (inductive) approach consulting with PwMS regarding their experiences of healthcare to correspond with the thematic analysis methodology.^16^ The research team's consumer reference group of PwMS was consulted during the design of interview questions and provided feedback on the consistent use of sensitive and inclusive language, and whether the questions were relevant and appropriate in the context of modern MS healthcare in Australia, such as the frequency and type of routine investigations and appointments with HCPs. For PwMS, the interview was divided into four broad areas: (1) Personal experience with MS, (2) Experience of diagnosis, (3) Experience of follow‐up appointments and (4) Experience with allied‐HCPs. For HCPs, the interview was divided into two broad areas: (1) Approach to diagnosis and (2) Approach to follow‐up appointments.

### 2.3 Analysis

Online questionnaires were downloaded into Microsoft Excel; we used descriptive statistics to describe the participant populations. Interviews were audio‐recorded, transcribed verbatim and analysed to identify themes. Analysis involved three coders from diverse backgrounds, including personal experience with MS, experience as an HCP and experience in coding qualitative data. Interviews were independently coded, with each coder identifying, analysing and interpreting the patterned response or themes and meaning in the data,^16^ with any coding discrepancies resolved via consensus. One coder used NVivo 12 software^17^ to collate the data and a second coder cross‐checked this coding.

## 3 RESULTS

### 3.1 Interview results

Fifteen PwMS (Table 1) and 11 HCPs (seven neurologists and four MS nurses) were interviewed. PwMS were located in the Australian Capital Territory (ACT) and Victoria, and the HCPs were from the ACT, New South Wales, Victoria and Western Australia.

**Table 1:** Demographics of PwMS, their clinical characteristics and healthcare teams

PwMS characteristics	*(n = 15)*
Mean age (years) (SD)	55.0 (11.1)
Mean age at diagnosis (years) (SD)	40.2 (7.9)
Gender [*n* (%)]	
Female	11 (73)
Male	4 (27)
Current MS classification [*n* (%)]	
Relapsing remitting	7 (47)
Secondary progressive	3 (20)
Primary progressive	2 (13)
Progressive relapsing	2 (13)
Unknown	1 (7)
Mean number of HCPs in the MS Care Team [*n* (SD)]	3.3 (1.9)
Types of HCPs from whom PwMS receive MS care^a^ [*n* (%)]	
Neurologist	12 (80)
GP	11 (73)
MS nurse	3 (20)
Allied‐healthcare professional	10 (67)
Types of allied‐HCPs from whom PwMS receive MS care^a^ [*n* (%)]	
Physiotherapist	4 (27)
Exercise physiologist	5 (33)
Massage therapist	2 (13)
Psychologist	1 (7)
Occupational therapist	1 (7)
Other	8 (53)

Abbreviations: GP, general practitioner; HCPs, healthcare professionals; MS, multiple sclerosis; PwMS, people living with MS.

^a^ Participants listed multiple HCPs in their healthcare team and many were seeing more than one allied‐HCP; therefore, percentages are greater than 100.

Following coding, three themes were common to both PwMS and HCPs: communication, uncertainty and relationship and rapport. Four unique themes were described by PwMS: decision‐making, roles and expectations, self‐directed management and support, and four unique themes were described by HCPs: medical management, multidisciplinary approach, barriers and access and hope and reassurance (Table 2).

**Table 2:** Identified themes for PwMS and HCPs

Shared themes for PwMS and HCPs	PwMS	HCPs
Communication	Decision‐making	Medical management
Uncertainty	Roles and expectations	Multidisciplinary approach
Relationship and rapport	Self‐directed management	Barriers and access
	Support	Hope and reassurance

Abbreviations: HCPs, healthcare professionals; PwMS, people living with MS.

### 3.2 Common themes for PwMS and HCPs

#### 3.2.1 Communication

Communication was a salient theme across all interviews. Both PwMS and HCPs stated that effective communication involved being clear and direct when delivering the diagnosis and discussing ongoing management.

He didn't beat around the bush [got straight to the point], he was very clear what was going on … he got across the message very quickly that what you want to do is get on some kind of immunomodulatory therapy as soon as you can. (PwMS02)

Demonstrate your findings to make it really clear that this is all based on objective findings and trying to use clear language, don't beat around the bush [get straight to the point] with the description or the language, if it's MS you need to say MS because that is what people are expecting to hear. (HCP04)

PwMS liked when their neurologist explained an examination or investigation to them, rather than just giving them the final diagnostic decision, with some PwMS expressing that they would like more of this practice.

He actually shows me the images as well, which is really great. (PwMS02)

I think if you've done a test, it's good to give the results and talk through them … that could definitely be done, and just a little more explaining and not assuming. (PwMS14)

For some PwMS, one of the most important aspects of clear, effective communication involved the use of simple language without using complex medical terminology. PwMS also stated that they would like clearer explanations and no assumption of prior knowledge, particularly during diagnosis.

The extremely good thing about the neurologist is that he will speak English and answer questions … not just instruct and speak jargon. (PwMS13)

He knew exactly what he was talking about and I didn't … it would have been great if he assumed I knew nothing. (PwMS14)

Several HCPs mentioned the importance of addressing any preconceived ideas of MS. These HCPs reported that most of their consultation time with a PwMS, both at the time of diagnosis and during follow‐up appointments, is spent providing education and correcting any misinformation.

Typically, in the modern world, they may access various information resources, many of which are misleading or, quite frankly, totally incorrect. That is quite common. I do give them what I believe are appropriate links to resources with authoritative and objective information, but not infrequently they still access, not incorrect but sometimes dangerous information. (HCP02)

One PwMS stated that they would like greater transparency when it comes to communicating the side effects of disease‐modifying therapies (DMTs).

You're told about the relapse reduction and all these great new drugs but you're not told of the side effects. (PwMS01)

Several HCPs noted that they generally do not discuss DMTs in the very early stages of diagnosis, explaining that they use a staged approach when communicating the diagnosis and management options, in an attempt to prevent overwhelming the PwMS with too much information.

We don't really even tend to touch on therapies in that really early diagnostic point. We let them know that there's therapies there, but we kind of save that for that second appointment, or for those that are still coming to terms with their diagnosis, that's in the third appointment … we're trying not to overload them with too much information all at once. (HCP05)

#### 3.2.2 Uncertainty

Uncertainty associated with MS was discussed in multiple interviews by both PwMS and HCPs. Three PwMS listed uncertainty as one of their three biggest concerns surrounding MS, either relating to the future of their progression or whether they were on a suitable treatment option.

The uncertainty of the future, you just don't know what's going to happen tomorrow, even if you're stable today. (PwMS15)

PwMS also said that they did not like it when their disease trajectory was framed as unpredictable or uncertain.

The overwhelming impression I got from him most of the time was that everything was completely unpredictable, so… take it one day at a time, and that wasn't as helpful. (PwMS02)

This notion of uncertainty was identified as a significant challenge for HCPs. Several neurologists said that the lack of definitive diagnostic tests for MS creates a level of uncertainty that can create difficulty in communicating an MS diagnosis.

I think it is important to discuss differential diagnosis … I never tell someone that it is one hundred percent certain that it is MS, because there is no absolute definitive pre‐mortem diagnosis apart from brain biopsy. (HCP04)

Adequate exploration of various differential diagnoses was highlighted by several HCPs as a way to mitigate this uncertainty. Uncertainty in disease trajectory also made it difficult for HCPs to provide reassurance or give an accurate prognosis when asked.

Very often people that are very, very emotional and distressed, it's a very difficult conversation to have and often people are just stressed because it's a fear of the unknown and they want someone to tell them that it's going to be okay and you can't do that. (HCP10)

#### 3.2.3 Relationship and rapport

The importance of developing rapport was a prominent theme discussed by both PwMS and HCPs. PwMS frequently reported that they liked it when their neurologist was engaged and showed an interest in them.

He was very sympathetic and he listened to what I had to say. (PwMS09)

Similarly, HCPs highlighted that building rapport included listening to the patient and directing care based upon their preferences and needs.

This is a good diagnosis in neurology because we've got lots of good treatment … so I start focussing on what's important to them so they know that the diagnosis is not the primary feature of their life … That's my approach to treating MS. (HCP06)

Whilst PwMS and HCPs shared the same belief in the importance of building rapport, relationship building was revealed to have multiple purposes for HCPs, which included building therapeutic alliances to facilitate treatment engagement.

So if you've commenced early and established effectively and the patient is tolerating and trusts you, trusts your judgement and your decision and is happy with the choice, then that's a big determinant of success. (HCP04)

Some PwMS said that they were not satisfied with their current relationship with their neurologist, with some describing disappointment as a result of poor rapport at the time of diagnosis.

There's a lot sort of lacking in the depth of the relationship… given that this is a life‐long thing, and it just always stays at that very superficial level. (PwMS01) They don't realise that this is now my whole world and to them, I'm just like a folder and they just open it, have a quick check, then close it. So, I felt very invalidated. (PwMS03)

### 3.3 Themes unique to PwMS

 

#### 3.3.1 Decision‐making

PwMS discussed that they would have liked greater guidance when it came to selecting a DMT and felt overwhelmed with the responsibility.

You're often just handed all this information and you've just received this huge life‐changing diagnosis and it's like ‘oh, you choose’, and that's quite overwhelming. (PwMS01)

Two PwMS also expressed the belief that they initially made the wrong DMT choice, and that this could have potentially been avoided if they had received more guidance from their neurologist.

I think I probably could have had eight months of my life back with much better quality, if I had just gone straight on Copaxone and if that had been adequately explained. (PwMS02)

He wasn't pushing it, he said it was really up to me and at the time, I really didn't want to do injections so I chose not to … and in retrospect it would have been good to go on them earlier. (PwMS11)

One PwMS outlined a positive decision‐making experience with his neurologist, where he felt that he had received sufficient information and guidance.

They were very good at talking about what treatment options there were, and what was better about each one and which would be the most preferable for me at the time. (PwMS13)

#### 3.3.2 Roles and expectations

PwMS reported a range of beliefs about the roles and expectations of their HCPs. Nine PwMS listed their neurologist as one of the most important members of their treatment team.

He has the ‘big guns’, because with the MS, he's the one who gives me the medication. (PwMS05)

Additionally, one person with primary progressive MS felt as though their neurologist could not offer them much in terms of management.

There's not much that he can do for me. We're just going to follow‐up every 12 months. (PwMS07)

Another PwMS explained that they had to change their expectations regarding their neurologist.

Once I noticed that my expectations were not being met, and that my expectations were probably very high and were probably never going to be met … as soon as I've dropped all of that I've been a lot happier. (PwMS01)

Some PwMS were uncertain as to what GPs could offer in terms of MS support and management.

I don't see what GPs can really do, it's a bit beyond them in that it's sort of specialised. (PwMS01)

However, GPs often played a more central role for PwMS in rural areas.

The GP can't initiate management when I've got an acute attack, I've got to see my neurologist who then has to arrange it, but my GP has been really good at triaging it so that I can actually have it done locally, because we're rural. (PwMS08)

#### 3.3.3 Self‐directed management

Multiple PwMS said that much of their disease and symptom management is instigated and directed by them. Some PwMS did not mind directing their own management and were happy to share the responsibility with their HCPs.

He said this is to the best of his knowledge what is available and if I felt that I was interested in anything else that I could research and I could discuss it with him if I wanted to. (PwMS10)

However, some PwMS were more averse to self‐directed management.

He didn't give me any information, I had to go and find it myself. (PwMS14)

#### 3.3.4 Support

Many PwMS reported inadequate emotional support from their HCPs at the time of diagnosis.

No one said to me, ‘I'm really sorry, this is what you've got’ … it's a very funny, lonely moment. (PwMS03)

The value of introducing access to support services early, including those provided by MS Australia and MS Limited, was discussed in numerous interviews.

I would have liked him to have referred me to the MS Society in the most strongest terms and say that they will help you with your immunotherapy, and don't attempt this without speaking to them. (PwMS02)

One participant expressed concern at a lack of government support towards MS.

I was knocked back by the NDIS and now I have to apply again … there is not enough government force and not enough support government wise. You are pretty much left on your own to struggle and it's on a daily basis. That's what they don't realise. You go home and live with it every day. (PwMS03)

### 3.4 Themes unique to HCPs

 

#### 3.4.1 Medical management

Optimal medical management of MS was discussed across all HCP interviews. HCPs discussed their approaches towards diagnosis, treatment, monitoring and ongoing care. When discussing diagnosis, many HCPs explained that they do not find classifying MS into specific phenotypes useful when communicating with PwMS, and prefer describing MS as active or nonactive.^12^

I think it is far more important now to call a patient active or non‐active, and that's the Lublin criteria which I think is much more helpful than the CIS, relapsing‐remitting, secondary progressive, primary progressive classification. (HCP01)

Some HCPs said they only consider these classical MS phenotypes as a requirement when prescribing medication from the Australian Pharmaceutical Benefits Scheme.

Additionally, HCPs highlighted the need for a personalized approach in MS that balances the factors and preferences of each individual, with the best available evidence, which is based on group outcomes.

There's no one size fits all, you really need to take a few things into account. (HCP02)

There are lots of options and they've all got a proven efficacy profile so they clearly all work for some people but you can't guarantee that they are all going to work for everyone so there is no absolute means of determining which is going to be the best tolerated treatment … it might depend on whether they are in the childbearing years and actively thinking about having a family in the near future or maybe you can already see that they're medication adverse and so you might move away from frequently administered treatments. (HCP04)

#### 3.4.2 Multidisciplinary approach

All HCPs described the importance of a multidisciplinary approach for MS healthcare. Some neurologists highlighted that neurology is becoming increasingly specialized.

Medicine is becoming more and more complicated, all of us only know a little slice of it, but we know that slice really well, and we're incredibly interested in optimising the outcomes for that slice. (HCP03)

Neurologists were described as playing a central and unique role within the multidisciplinary team, typically with a greater focus on the treatment and prevention of relapses (due to their ability to prescribe DMTs), while nurses and other allied‐health professionals were often more focused on everyday functioning and quality of life. It was also stated that as the severity of MS increases, the engagement of a comprehensive multidisciplinary team becomes more important.

The people with advanced MS are better managed by a multidisciplinary team, because their management is dominated by their physical demands … They should, however, still have some contact with a neurologist, to cope with neurology‐specific things. (HCP02)

Multiple neurologists also explained the usefulness of having an MS nurse to assist in the management of their patients.

Having a nurse there is essential. (HCP01)

#### 3.4.3 Barriers and access

Many barriers and issues in access to healthcare were identified by HCPs. One commonly identified barrier for neurologists was time constraints and availability of appointments, particularly in the public system.

Time constraints … twenty minutes is often not ideal and in private practice people might be able to offer more than what we can in the public sector, and again the ability to see people quickly. (HCP07)

Reduced access to MS specialists (both nurses and neurologists) in rural areas was identified as a barrier to care.

For some patients, access to neurologists is very difficult … Our average waiting time is six months, so I'm trying to get these patients in early if they get referred to me, but others might wait six months. (HCP01)

A lack of funding was discussed by several HCPs.

I don't think the health system in Australia recognises and funds that clinical nursing consultant role the way it should, particularly in chronic disease … MS is a great example of a chronic disease where the input of a nurse can be really critical. (HCP04)

The government is not funding enough specialised clinics. (HCP01)

#### 3.4.4 Hope and reassurance

Hope and reassurance was identified as a theme, often discussed in the context of communication. Many HCPs said that they focus on delivering hope to their patients, often through highlighting advances in MS therapies, and how a diagnosis of MS today carries a much more positive prognosis than it did in the past.

I point out in the last five years we've had more advances than in the last twenty. So, I stress the positive aspect, and I also stress that science has the explanation. I point out that the majority of new lesions do not produce any symptoms. (HCP02)

I think its positivity and a plan are the two most important things. I think being realistic is important too. But I think in this day and age reality and optimism go together because we've got options. You can be realistic and optimistic. (HCP06)

## 4 DISCUSSION

In this study, we found similarities and differences in the experiences of healthcare for PwMS and their HCPs. Both groups valued clear communication, recognized the uncertainties associated with MS diagnosis, treatment and progression and highlighted the importance of developing rapport in this dyadic relationship. These common themes are interrelated, with both groups recognizing that effective communication is integral in developing rapport, and that uncertainty can present as a barrier in this process. However, the purpose of developing rapport was multifaceted and not necessarily consistent between the two groups. For example, some PwMS wanted to be supported through a sense of connection with their HCPs. This was also valued by some HCPs; however, HCPs also recognized the importance of relationship and rapport in developing therapeutic alliance, trust and treatment adherence. A recent literature review supports this rationale held by HCPs, that effective communication can enhance the healthcare experience for PwMS, improve treatment adherence and lead to better patient outcomes.^18^

In this study, communication was the salient theme across all interviews with both PwMS and HCPs and is clearly a strong determinant of the healthcare experience. Many of the issues identified by PwMS were associated with a lack of open and empathetic communication with their HCPs. Overall, PwMS wanted support and assistance with their decision‐making from their HCPs. PwMS often undergo substantial self‐directed efforts to receive enough information to meet their needs.^9^ In this study, the level of need for self‐directed management was variable across PwMS and was often linked to their understanding of the roles and their expectations of the HCPs. To overcome this, some PwMS said they changed their expectations and sought support elsewhere, whilst others said that they felt disappointed when their HCPs did not meet their expectations and needs.

**Figure 1:**
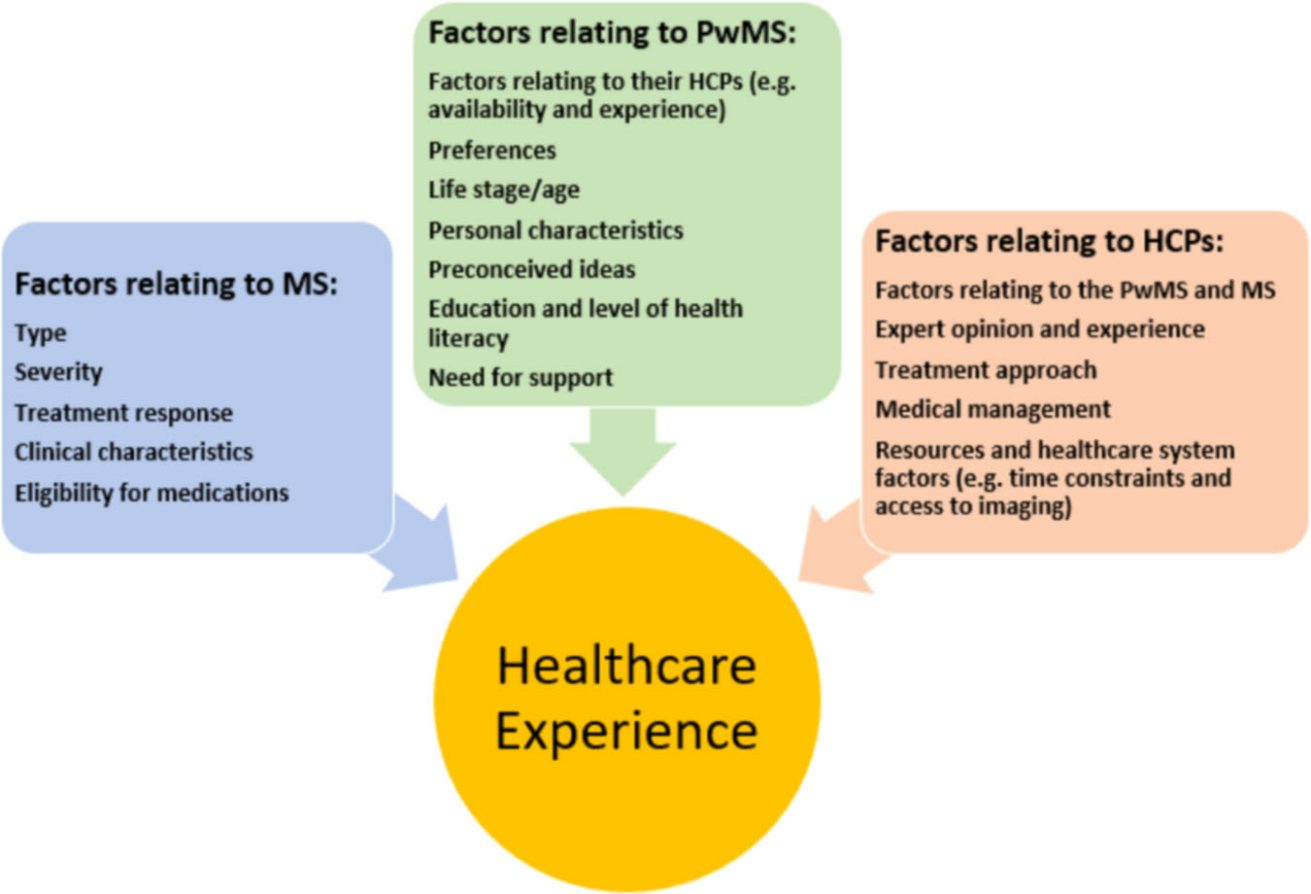
Factors relating to MS, PwMS and HCPs jointly contribute to the experience of healthcare. HCPs, healthcare professionals; MS, multiple sclerosis; PwMS, people living with MS

Modern management of MS presents many challenges for HCPs and demands consideration of many factors, as indicated in Figure 1. Treating neurologists need to consider uncertain drug efficacy and disease trajectory,^10^ as well as potential side effects and financial impact,^11^ and this is often in the context of time constraints and limited resources. This study highlighted that HCPs' perspectives and experiences of healthcare were guided by factors that were often not transparent to PwMS. HCPs aimed to provide hope and reassurance whilst focusing on delivering healthcare that is both evidence‐based and individualized, to achieve optimal health outcomes. Neurologists also considered factors associated with a complex health system (e.g., barriers and access) whilst often leading a multidisciplinary team of HCPs. The lack of transparency to PwMS of the multiple factors considered by HCPs may be contributing to the experiences of PwMS of poor communication, confusion around the roles and expectations of HCPs, experiences of a superficial relationship with their HCPs and low support with decision‐making.

Discrepancies between PwMS and HCPs in perspectives and expectations of care have been documented previously.^6^, ^12^ The need for improved communication is consistent with the existing literature,^13^ as well as the need for adequate empathy, support and person‐centred care.^19^ Therefore, clear communication of factors that impact the healthcare experience in MS, as shown in Figure 1, may provide a more positive experience for both PwMS and HCPs and contribute to optimal health outcomes. Figure 1 outlines that the healthcare experience is guided by multiple factors that relate to the MS presentation, the PwMS and the HCP. Whilst both groups recognize factors associated with the PwMS (preferences, age), MS presentation (type, severity, symptoms) and the HCP (preferences, treatment approach, experience), the HCP is also considering additional factors that can influence and complicate management (optimal medical management, barriers, resources) and these may not be clearly communicated to the PwMS. This may explain why some PwMS feel as though their concerns have not been received by their HCPs when they are presented with management options that do not align with their preferences. It is known that alignment of appropriate management with personal preferences and goals of the PwMS can present as a challenge for neurologists.^11^ It can be difficult for neurologists to elicit PwMS' preferences and goals amidst the stress surrounding diagnosis.^20^ Some studies have shown that patient decision aids can help to share the decision‐making process and ensure that PwMS' preferences are met during DMT selection.^20^, ^21^

When communicating with PwMS, HCPs reported that they focus on addressing misinformation and then temporally staging their discussion of diagnosis and treatment across multiple consultations to prevent overwhelming the PwMS with too much information at an emotionally distressing time. However, this staged approach to communication was at times interpreted by PwMS as the HCP not providing adequate assistance with decision‐making and a lack of empathy that impacted rapport development. It may be useful for HCPs to comprehensively explain the rationale for their staged‐communication approach to provide reassurance and support with decision‐making to PwMS, which may build trust and therapeutic alliance.

### 4.1 Strengths and limitations

One major strength of the study was the semi‐structured interview format, allowing PwMS to openly share their diverse perspectives and experiences of healthcare. Participants were from various regions across Australia, providing greater generalizability. However, not all regions were included, and differences between urban and rural areas could be examined further. The study was limited by a small sample size and thus findings were not stratified according to demographic data. Additionally, the perceptions of PwMS who have minimal interaction with HCPs may not have been captured comprehensively in this study. It should be noted that healthcare experiences of PwMS may vary greatly depending on the year in which they were diagnosed with MS, and the study population was fairly heterogeneous regarding time of diagnosis. In the past 10 years, there has been a significant increase in the number of DMTs available. Therefore, it is possible that PwMS who were diagnosed more recently were more hopeful and had a less negative experience than those diagnosed before this time.

## 5 CONCLUSION

Many factors are considered by PwMS and HCPs during their interactions. However, the multiple factors considered by HCPs that guide their decisions are not always transparent to PwMS and could be better communicated. During interactions with PwMS and HCPs, PwMS often focus on their unique personal factors and factors relating to their MS. In the same consultation, HCPs consider those factors as well as factors associated with optimal medical management, the health system and working in a multidisciplinary team, which is not always transparent or communicated clearly to the PwMS. Many of the issues with MS healthcare identified by PwMS, such as lack of support and assistance with decision‐making, could be resolved by more open and empathetic communication with their HCPs. HCPs could also explain the rationale for taking a staged approach when communicating the diagnosis and management of MS, which may improve therapeutic alliance and provide reassurance for PwMS about this approach. To give PwMS realistic expectations of what their HCPs can provide, HCPs should clearly outline the role that they will play in MS management, as well as the roles that can be played by other allied‐HCPs and MS support organizations. These interactions may improve healthcare experiences of both PwMS and HCPs, leading to better engagement with treatment and better overall health outcomes.

## CONFLICT OF INTERESTS

The authors declare that there are no conflicts of interest.

## Data Availability

Data will be made available from the corresponding author upon reasonable request.
